# Supplementary material for: Slower growth of Escherichia coli leads to longer survival in carbon starvation due to a decrease in the maintenance rate
Source: Mol Syst Biol. 2020 Jun 5;16(6):e9478. doi: 10.15252/msb.20209478 (PMC7273699; doi:10.15252/msb.20209478)
Supplement: Supplementary file 1 — Expanded View Figures PDf [file MSB-16-e9478-s001.pdf]

Expanded View Figures

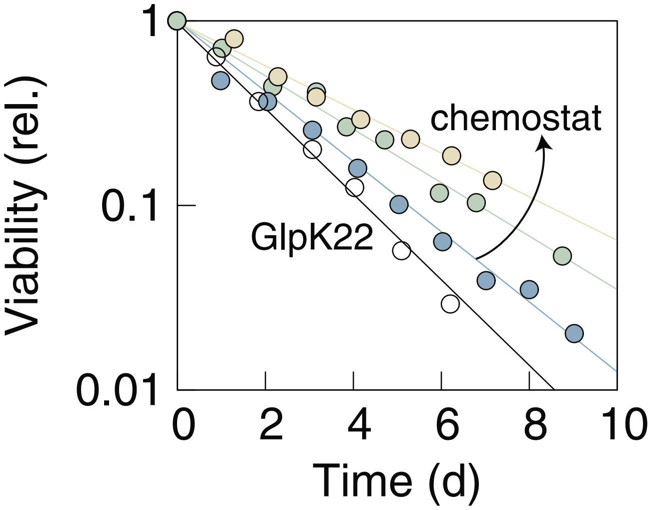

Figure EV1. Survival kinetics after different growth conditions.
Wild-type *Escherichia coli* K-12 are starved after growth in chemostat at growth rates $\mu = 0.3{,}0.5{,}0.7\ h^{-1}$ (colored data). GlpK22 mutants are starved after growth in batch at $\mu = 0.9\ h^{-1}$ (white circles). Colors correspond to the ones in the legend of Fig 2. Experimental data are shown normalized to the highest viability value at the beginning of starvation. All the cultures die exponentially, spanning a range from 0.25 day^{-1} (WT previously grown at 0.3 h^{-1}, yellow circles) to 0.59 day^{-1} (GlpK22, previously grown at 0.9 h^{-1}, white circles).

 © 2020 The Authors
