# Supplementary material for: Slower growth of Escherichia coli leads to longer survival in carbon starvation due to a decrease in the maintenance rate
Source: Mol Syst Biol. 2020 Jun 5;16(6):e9478. doi: 10.15252/msb.20209478 (PMC7273699; doi:10.15252/msb.20209478)
Supplement: Supplementary file 2 — Table EV1 [file MSB-16-e9478-s002.docx]

| Strain | Growth mode | Carbon source | Growth rate  $\mu{(h}^{-1})$ | Death rate  $\gamma{(d}^{-1})$ |
| --- | --- | --- | --- | --- |
| WT | batch | LB | 2.00 ± 0.02 | 1.31 ± 0.09 |
| WT | batch | CAA+Glucose | 1.31 ± 0.04 | 0.64 ± 0.15 |
| WT | batch | CAA+Glycerol | 1.20 ± 0.03 | 0.64 ± 0.11 |
| WT | batch | Glucose | 0.92 ± 0.03 | 0.48 ± 0.04 |
| WT | batch | Xylose | 0.75 ± 0.02 | 0.46 ± 0.02 |
| WT | batch | Glycerol | 0.70 ± 0.02 | 0.43 ± 0.02 |
| WT | batch | Succinate | 0.69 ± 0.02 | 0.43 ± 0.03 |
| WT | batch | Acetate | 0.42 ± 0.04 | 0.35 ± 0.05 |
| WT | batch | Mannose | 0.29 ± 0.05 | 0.28 ± 0.02 |
| WT | batch | Proline | 0.27 ± 0.04 | 0.32 ± 0.02 |
| WT | batch | Glutamate | 0.13 ± 0.01 | 0.27 ± 0.02 |
| WT | chemostat | Glycerol | 0.70 ± 0.04 | 0.40 ± 0.04 |
| WT | chemostat | Glycerol | 0.50 ± 0.03 | 0.33 ± 0.03 |
| WT | chemostat | Glycerol | 0.40 ± 0.02 | 0.30 ± 0.03 |
| WT | chemostat | Glycerol | 0.30 ± 0.02 | 0.29 ± 0.03 |
| WT | chemostat | Glycerol | 0.20 ± 0.01 | 0.26 ± 0.03 |
| WT | chemostat | Glycerol | 0.10 ± 0.01 | 0.24 ± 0.02 |
| GlpK22 | batch | Glycerol | 0.90 ± 0.02 | 0.59 ± 0.03 |

**Table EV1. Growth and death rates of wild type cells and GlpK22 mutants.** Growth and death rates of wild type cells (WT) and GlpK22 mutants (NQ898) grown in batch or continuous cultures in minimal medium supplemented with different carbon sources. “CAA” denotes casamino acids Unless otherwise noted, growth and death rates are averages of three independent experimental repeats, reported with one standard deviation. Uncertainties of growth rates in the chemostat are reported with estimated error of 5%, due to the feeding pump. Note that the wild type culture grown at $0.7 h^{-1}$ in the chemostat shows a death rate of $\gamma_{\mu=0.7/h,}=0.40\pm0.04 d^{-1}$, which is in good agreement with the batch culture value $\gamma_{\mathrm{batch}}=0.43\pm0.02 d^{-1}$, where cells also grow at a similar growth rate.
