# Supplementary material for: Slower growth of Escherichia coli leads to longer survival in carbon starvation due to a decrease in the maintenance rate
Source: Mol Syst Biol. 2020 Jun 5;16(6):e9478. doi: 10.15252/msb.20209478 (PMC7273699; doi:10.15252/msb.20209478)
Supplement: Supplementary file 3 — Table EV2 [file MSB-16-e9478-s003.docx]

| Strain | Growth  mode | Recycling  yield | Maintenance rate  ($\mathrm{fmol}\mathrm{CFU}^{-1} d^{-1}$) |
| --- | --- | --- | --- |
| WT | Chemostat – 0.1/h | 0.12 ±0.01 | 0.16 ± 0.01 |
| WT | Chemostat – 0.3/h | 0.14 ± 0.01 | 0.27 ± 0.02 |
| WT | Chemostat – 0.5/h | 0.18 ± 0.01 | 0.37 ± 0.02 |
| WT | Chemostat  – 0.7/h | 0.19 ± 0.02 | 0.50 ± 0.02 |
| WT | batch | 0.19 ± 0.02 | 0.59 ± 0.04 |
| GlpK22 | batch | 0.23 ± 0.02 | 0.89 ± 0.04 |

**Table EV2. Recycling yield and maintenance rate.** Recycling yield and maintenance rate values of wild type *E.* *coli* K-12 (WT) and GlpK22 mutants (NQ898) grown in batch or continuous cultures in minimal medium supplemented with glycerol. Relative growth rates and death rates are reported in Table EV1. Yield and maintenance values are obtained from a least square fit to absolute regrowth and lag times, as shown in Fig. 2 and are reported with one standard deviation.
