# Supplementary material for: Slower growth of Escherichia coli leads to longer survival in carbon starvation due to a decrease in the maintenance rate
Source: Mol Syst Biol. 2020 Jun 5;16(6):e9478. doi: 10.15252/msb.20209478 (PMC7273699; doi:10.15252/msb.20209478)
Supplement: Supplementary file 4 — Table EV3 [file MSB-16-e9478-s004.docx]

| Strain | Growth  mode | Average length  ($\mu$m) | Average width  ($\mu$m) | Average volume  ($\mu m^{3}$) | Recycling  yield per volume  ($\mu m^{-3}$) | Maintenance  rate per volume ($\mathrm{fmol}\mathrm{CFU}^{-1}$ $d^{-1}\mu m^{-3}$) |
| --- | --- | --- | --- | --- | --- | --- |
| WT | Chemostat – 0.1/h | 1.57 | 0.65 | 0.45 | 0.28 ± 0.03 | 0.36 ± 0.03 |
| WT | Chemostat – 0.3/h | 1.59 | 0.69 | 0.52 | 0.27 ± 0.02 | 0.52 ± 0.04 |
| WT | Chemostat – 0.5/h | 1.72 | 0.77 | 0.69 | 0.26 ± 0.02 | 0.53 ± 0.04 |
| WT | Chemostat  – 0.7/h | 1.80 | 0.80 | 0.77 | 0.25 ± 0.03 | 0.64 ± 0.04 |
| WT | batch | 1.83 | 0.81 | 0.80 | 0.24 ± 0.02 | 0.74 ± 0.06 |
| GlpK22 | batch | 1.85 | 0.83 | 0.86 | 0.26 ± 0.03 | 1.02 ± 0.07 |

**Table EV3.** **Recycling yield and maintenance rate per cell volume.** Recycling yield and maintenance rate per cell volume values reported with relative length, width and volume of wild type cells (WT) and GlpK22 mutants (NQ898) grown in batch or continuous cultures in minimal medium supplemented with glycerol. Relative growth rates and death rates are also reported (see also Table EV1). Cell length and width values are averages of 200 cells. Cell volume is calculated from length and width as described in Methods. We estimate that cell volumes are determined only to about 5%, due to limitations of phase contrast microscopy. Uncertainties of cell volume measurements are propagated into recycling yield and maintenance rate per volume. Values from yield and maintenance values are shown in Table EV2.
