# Supplementary material for: Slower growth of Escherichia coli leads to longer survival in carbon starvation due to a decrease in the maintenance rate
Source: Mol Syst Biol. 2020 Jun 5;16(6):e9478. doi: 10.15252/msb.20209478 (PMC7273699; doi:10.15252/msb.20209478)
Supplement: Supplementary file 5 — Table EV4 [file MSB-16-e9478-s005.docx]

| Strain | Growth mode | Carbon source | Growth rate  $\mu{(h}^{-1})$ | Death rate  $\gamma{(d}^{-1})$ |
| --- | --- | --- | --- | --- |
| $\Delta$rpoS | batch | LB | 2.00 ± 0.07 | 1.64 ± 0.09 |
| $\Delta$rpoS | batch | CAA + Glucose | 1.32 ± 0.01 | 0.78 ± $0.08$ |
| $\Delta$rpoS | batch | CAA + Glycerol | 1.15 ± 0.04 | 0.82 ± $0.08$ |
| $\Delta$rpoS | batch | Glucose | 0.82 ± 0.03 | 0.82 ± 0.01 |
| $\Delta$rpoS | batch | Xylose | 0.73 ± 0.03 | 0.73 ± $0.03$ |
| $\Delta$rpoS | batch | Glycerol | 0.70 ± 0.04 | 0.76 ± $0.05$ |
| $\Delta$rpoS | batch | Succinate | 0.73 ± 0.02 | 0.69 ± $0.05$ |
| $\Delta$rpoS | batch | Acetate | 0.44 ± 0.07 | 0.77 ± $0.18$ |
| $\Delta$rpoS | batch | Mannose | 0.38 ± 0.11 | 0.57 ± $0.04$ |
| $\Delta$rpoS | batch | Proline | 0.34 ± 0.04 | 0.68 ± $0.16$ |
| $\Delta$rpoS | batch | Glutamate | 0.13 ± 0.03 | 0.46 ± $0.05$ |

**Table EV4. Growth and death rates of** $\boldsymbol{\Delta}$**rpoS mutants grown in different media.** Growth and death rates of $\Delta$rpoS mutants grown in batch cultures in minimal medium supplemented with different carbon sources. Growth rate and death rate values are averages of three independent experimental repeats, reported with one standard deviation. “CAA” denotes casamino acids.
